# Adult Rat Bones Maintain Distinct Regionalized Expression of Markers Associated with Their Development

**DOI:** 10.1371/journal.pone.0008358

**Published:** 2009-12-21

**Authors:** Simon C. F. Rawlinson, Ian J. McKay, Mandeep Ghuman, Claudia Wellmann, Paul Ryan, Saengsome Prajaneh, Gul Zaman, Francis J. Hughes, Virginia J. Kingsmill

**Affiliations:** 1 Institute of Dentistry, Barts & The London School of Medicine and Dentistry, Queen Mary University of London, London, United Kingdom; 2 Dental Institute, Kings College London, Guy's Hospital, London, United Kingdom; 3 Department of Veterinary Basic Sciences, The Royal Veterinary College, London, United Kingdom; Ecole Normale Supérieure de Lyon, France

## Abstract

The incidence of limb bone fracture and subsequent morbidity and mortality due to excessive bone loss is increasing in the progressively ageing populations of both men and women. In contrast to bone loss in the weight-bearing limb, bone mass in the protective skull vault is maintained. One explanation for this could be anatomically diverse bone matrix characteristics generated by heterogeneous osteoblast populations. We have tested the hypothesis that adult bones demonstrate site-specific characteristics, and report differences at the organ, cell and transcriptome levels. Limb bones contain greater amounts of polysulphated glycosaminoglycan stained with Alcian Blue and have significantly higher osteocyte densities than skull bone. Site-specific patterns persist in cultured adult bone-derived cells both phenotypically (proliferation rate, response to estrogen and cell volumes), and at the level of specific gene expression (collagen triple helix repeat containing 1, reelin and ras-like and estrogen-regulated growth inhibitor). Based on genome-wide mRNA expression and cluster analysis, we demonstrate that bones and cultured adult bone-derived cells segregate according to site of derivation. We also find the differential expression of genes associated with embryological development (Skull: *Zic*, *Dlx*, *Irx*, *Twist1* and *Cart1*; Limb: *Hox*, *Shox2*, and *Tbx* genes) in both adult bones and isolated adult bone-derived cells. Together, these site-specific differences support the view that, analogous to different muscle types (cardiac, smooth and skeletal), skull and limb bones represent separate classes of bone. We assign these differences, not to mode of primary ossification, but to the embryological cell lineage; the basis and implications of this division are discussed.

## Introduction

In weight-bearing bones, a mechanically-driven homeostatic feedback mechanism is used to achieve a ‘target strain’ level and ensures that bone strength is maintained to resist fracture [1], [2]. This mechano-adaptive mechanism can fail, and when bone mass no longer matches mechanical demands, fractures are likely to ensue. Questions generally address ‘*why is bone mass lost from weight-bearing limb bones despite continued usage*?’ However, in parietal bones of the skull the levels of strain are low (low enough to induce ‘disuse’ bone loss in the limb) yet paradoxically, skull bones attain mechanical competence and resist potentially catastrophic levels of osteopenia despite this dramatic difference in the local mechanical environment [3], [4]. Therefore, it might be better to instead frame the question in a different manner and ask ‘*how is bone mass and mechanical competence achieved in low weight-bearing skull bones despite continued ‘disuse’?*’ Addressing the latter question leads to a conceptually novel approach to understand the attainment of mechanical competence in limb bones.

Several lines of evidence have suggested persistent differences in the adult bones derived from the distinct primary ossification processes. In humans and rodents, the mineral density and calcium concentration is greater in the skull than in the post cranial skeleton [5]–[9]. Osteocytes of mouse skull vault calvarial bones have a rounded appearance compared with the more elongated osteocytes of long bone fibulae [10]. Matrix composition differences have been described [11] and demineralised powder from skull bone does not induce bone formation by an endochondral ossification mode as limb bone derived powders [12]. Mechanical strain (ε) is the ratio of the change in length (new length (L′) minus original length (L)) divided by L, is defined as: (L′–L)/L and thus, has no units. A positive strain represents stretching, whilst compression is represented by a negative figure. Direct experimental measurements in a human showed that the strain levels in the parietal bone of the skull are 10 fold lower compared with the tibia [4]. We have previously demonstrated peak surface strains on the rat parietal bone of only 30 µε [3] – and that such bones are not mechanically responsive [3], [13]. If limb bones, that normally experience habitual functional strains ranging between 1800–3200 µε [14], were subject to such low mechanical strain levels, significant bone loss would ensue. Intriguingly, parietal bones of the skull vault do not appear to be subject to such ‘disuse’ bone loss [15]–[17]. The skull bones are also resistant to post-menopausal hormonal changes or glucocorticoid treatment-induced osteoporosis. Clinically, autogenous bone grafting using blocks of intramembranous bone resorb less than bone derived from endochondral sites [18]–[20].

These differences may be a consequence of the site-specific differences in osteoclast activities [21]–[23], however the observations also imply that the mechanisms regulating site-specific osteoblast behaviour/matrix production are autonomous. The basis of this independence, and especially the intrinsic characteristics of skull and limb osteoblasts, has not, to our knowledge, been fully assessed. We suggest that cell lineage, where limbs develop from lateral plate mesoderm and the skull from cranial neural crest [24], may in part contribute to such autonomy. The existence of lineage based differences in the skeleton is supported by specific developmental defects that are restricted to different skeletal compartments. Disruption of the TBX family of mesoderm transcription factors affects limb development (*TBX5*; Holt-Oram syndrome, *TBX3*; Ulnar-mammary syndrome [25], [26]) whereas *Cart1* knockout mice are born without a cranium [27], *Dlx5^−/−^* mice are born with abnormal osteogenesis of the skull vault and delayed cranial ossification, whilst the limbs show no obvious defects [28].

We postulated that skull (S) and limb (L) bones of the adult, and the bone-derived cells (Bdc) isolated from them (S-Bdc and L-Bdc) would have distinct transcriptomes reflecting specific origins. We have, therefore, investigated basal gene expression patterns by genome-wide microarray analysis. The consequence of any such transcriptome differences may be manifest as characteristic local osteoblast behaviour; therefore, we have studied bone matrix composition and the effect of ovariectomy on osteocyte number *in vivo*. We have also compared the *in vitro* characteristics of isolated S-Bdc and L-Bdc, testing whether they differ with respect to proliferation rates and cell volume. We show site-specific differences in the cellular and material composition of bone organs, and patterns of basal gene expression in skull and limb bones and adult bone-derived cells. In culture, the differential gene expression continues to reflect their site of origin, and the phenotypes of S-Bdc and L-Bdc differ. Surprisingly, we also note the persistent expression of site-specific markers associated with development of the skeleton in both bone organ and isolated adult bone-derived cells.

## Results

### mRNA expression in bone organs

**Figure 1:**
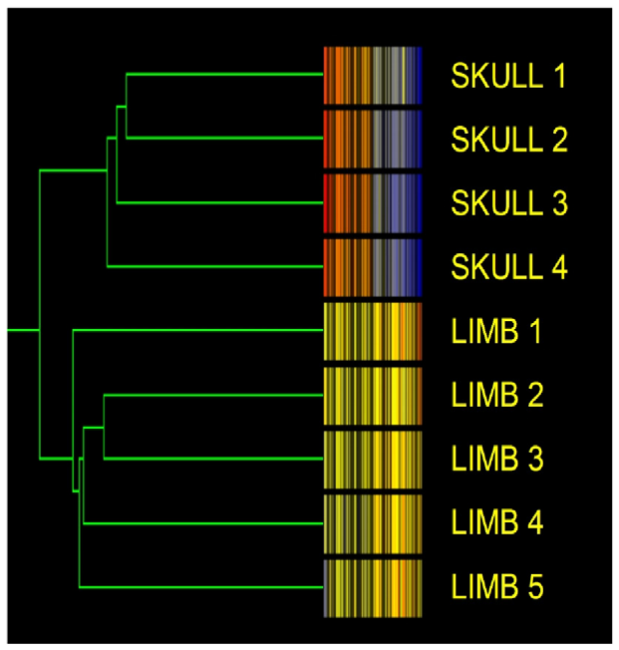
Bones from functionally different skeletal sites represent biologically distinct populations. Dendrogram generated by gene tree clustering analysis in GeneSpring 6.1 comparing basal expression of all filtered genes in skull and limb bone organs. Colours represent those genes (1236 of the genome) having expression levels with at least a significant, two-fold difference.

1236 genes (approximately 4% of the genome) were found to be significantly and, at least two-fold differentially expressed between ulnar limb and parietal skull bone. Cluster analysis segregated the two sites into distinct populations (Figure 1).

We selectively searched for those differentially expressed genes previously associated with the ossification process, Wnt signalling, developmental patterning and osteoporosis. Polymorphisms of a number of genes differentially expressed in our rat bone array have been associated with susceptibility to human osteoporosis [29]–[31]: *Opg* (skull∶limb, fold increase 3.1), *Vdr* (4.8), *Pthr1* (2.9), *Calcr* (4.6), *Lrp5* (2.2), and *Ctsk* (2.7), *Alox12* (0.2), Table 1. Cathepsin K, is generally considered as a marker for osteoclasts. Therefore, the increased expression detected in the skull samples may reflect a difference in osteoclast numbers at this site. This difference, however, is lost in the osteoblast cultures (see below).

**Table 1:** Differentially expressed genes in skull and limb bone associated with developmental patterning, Wnt signalling, osteoporosis and ossification.

High expression in skull	Gene Symbol	S∶L ratio	Gene Symbol	S∶L ratio	High expression in limb	Gene Symbol	S∶L ratio
	Sfrp2	13.51	Alcam	2.58		Btg2	0.49
	Cart1	10.76	Eln	2.57		Grpca	0.49
	Sost	5.36	Prelp	2.54		Svil	0.48
	Amelx	5.12	Twist1	2.51		Lect2	0.43
	Vdr	4.81	Sema5a	2.49		Best5	0.38
	Calcr	4.66	Bmpr2	2.48		Comp	0.38
	Ambn	4.23	Sp7	2.42		Mmp8	0.29
	Wif1	4.17	Ank	2.37		Pdlim7	0.24
	Gpnmb	3.81	Lrp4	2.35		Bmp5	0.22
	Cd276	3.74	Mmp13	2.32		Ctnnb1	0.20
	Igfbp5	3.68	Nab2	2.26		Wnt16	0.06
	Tcf7	3.51	Dlx5	2.22		Hoxa5	0.05
	Bmp3	3.43	Ptprv	2.20		Shox2	0.05
	Bmp6	3.29	Enpp1	2.19		Cacna1s	0.04
	Acp5	3.28	Tpp1	2.19		Csrp3	0.04
	Nog	3.24	Cdh15	2.17			
	Frzb	3.14	Dlx3	2.16			
	Cyp26b1	3.04	Lrp5	2.15			
	Tnfrsf11b	2.93	Ptn	2.13			
	Calca	2.92	Cpz	2.09			
	Pthr1	2.91	Ibsp	2.09			
	Csf1r	2.89	Omd	2.08			
	Cthrc1	2.83	Fgfr1	2.06			
	Csf1	2.82	Tcf3	2.04			
	Plau	2.81	Mitf	2.01			
	Mmp9	2.78	Igf2bp2	2.00			
	Ctsk	2.70					

S∶L ratio  =  Skull∶Limb ratio.

The differential expression of transcription factors (Table 2) between distinct anatomical bone locations may account for the site-specific mineralization levels, underlie their differential susceptibility to pathological osteopenic changes, and also be central to specification.

**Table 2:** Differentially expressed transcription factors in skull and limb bone.

Gene Symbol	S∶L ratio	Gene Symbol	S∶L ratio	Gene Symbol	S∶L ratio	Gene Symbol	S∶L ratio
Meis2	31.07	Nfib	2.52	Kpna1	0.50	Sox6	0.32
Nr2f1	11.02	Sox17	2.51	Solt	0.50	Tal1	0.32
Cart1	10.76	Twist1	2.51	Btg2	0.49	Gfi1b	0.31
Zic3	10.15	Lztr2	2.49	Rab8b	0.49	Trib3	0.31
Rfx4	8.56	Rai14	2.47	Chd4	0.48	Hoxc5	0.30
Zic4	8.13	Maf	2.40	E2f8	0.48	Gata1	0.29
RGD1311558	4.98	Tbx2	2.39	Gata3	0.48	Klf1	0.28
Wnk4	4.84	Satb2	2.35	Mafg	0.47	Padi4	0.28
Vdr	4.81	Kcnh2	2.27	Mafk	0.47	Ppp1r12b	0.27
Zic1	4.26	Nab2	2.26	Trak2	0.47	Trim29	0.27
Pax8	4.06	RGD1565031	2.25	Abtb1	0.46	Unr	0.27
Fbxl22	3.73	Dlx5	2.22	Cebpe	0.45	Ankrd1	0.26
Tcf7	3.51	LOC685277	2.20	Klf5	0.45	Hand2	0.26
Hdac10	3.40	Nr2f2	2.18	Tcfdp2	0.45	Fhdc1	0.25
Cdkn2a	3.31	Dlx3	2.16	Ybx2	0.45	Hoxb8	0.25
Prdm1	3.04	Mxd4	2.15	Creg	0.44	Thrsp	0.23
C2ta	2.92	Nfatc4	2.13	Tbx15	0.44	Bach1	0.22
Creb3l1	2.92	Rai14	2.12	Cited4	0.42	Dmrt2	0.21
Jundp2	2.89	Zfhx3	2.11	Sec14l2	0.42	Smyd1	0.21
Zfhx4	2.78	RGD1305899	2.05	Mlf1ip	0.41	Ctnnb1	0.20
Flywch1	2.68	Tcf3	2.04	R1b	0.41	Hoxa7	0.19
Pde8a	2.68	Epas1	2.03	Zfp278	0.40	RGD1561431	0.15
Fos	2.67	Hes1	2.01	Hipk3	0.37	RGD1566402	0.12
Maged1	2.63	Mitf	2.01	Asb1	0.34	Shox2	0.11
Armc9	2.57	Zfp98	2.01	Centg3	0.34	Tbx5	0.08
Supt3h	2.56			Nfe2	0.34	Hoxa10	0.06
Npas3	2.54			Zfp207	0.34	Hoxa5	0.05
Uncx4.1	2.54			Phox2a	0.32		

S∶L ratio  =  Skull∶Limb ratio.

Markers for homeobox genes associated with embryological development and body patterning are expressed site specifically in adult bones (Table 3. In addition, our preliminary data using adult mice bones are also presented in this table, and demonstrates positional identity marker expression, at least, appears to be conserved between these two species).

**Table 3:** Differentially expressed genes in rat and mouse bone associated with embryological development and body patterning.

Skull∶Limb ratio	GENE	RAT	MOUSE		RAT	MOUSE
	Hoxa3		2.33	Skull only	Hoxa2	Phox2a
	Hoxa5	0.05	0.12		Hoxd3	Tbx18
	Hoxa7	0.19	0.17		Irx1	Tbx19
	Hoxa10	0.06	0.14		Irx2	
	Hoxa11		0.15		Irx4	
	Hoxa11s		0.04		Twist2	
	Hoxb7	0.41			Msx2	
	Hoxb8	0.25			Tbx1	
	Hoxc5	0.30			Tbx3	
	Hoxc6		0.30		Runx1	
	Hoxd8		0.27	Limb only	Hoxc9	Tcfe3
	Cart1	10.76	4.55/3.65		Tcfe3	
	Dlx1		2.21/0.31			
	Dlx3	2.16	2.38			
	Dlx5	2.22	2.60			
	Msx1		3.19/0.44			
	Msx2		2.96			
	Phox2a	0.31				
	Shox2	0.08/0.14	0.06/0.18			
	Tbx2	2.36/2.4	2.75			
	Tbx3		0.32			
	Tbx5	0.07				
	Tbx14		0.06			
	Tbx15	0.44	0.36			
	Tcf3	2.04	2.07			
	Tcf4		0.20			
	Tcf7	3.51				
	Tcfcp212		2.91			
	Twist1	2.31/2.70	11.49			
	Twist2		0.34			
	Zic1	4.26	25.80			
	Zic2		12.53			
	Zic3	8.66	24/4.79			
	Zic4	8.13	5.20/4.34			
	Zic5		9.32/6.65			

Some genes are represented with more than one probe set on the array, therefore some genes have two values.

In work published by Xing *et al*., *in vivo* mechanical loading induced significant changes in the expression levels of genes in loaded limbs compared with control (unloaded) limbs [32]. We have compared their published set of mechanically responsive genes with our set of basal, site-specific differentially expressed genes. The common genes from these two data sets are presented in Table 4. It is apparent that some of the genes whose expression is stimulated by osteogenic mechanical loading are also constitutively expressed more highly in skull bone.

**Table 4:** Genes induced by loading in the limb (*) and differentially expressed basally between skull and limb bone. (*) Xing et al. JCB 96:1049–1060 (2005).

Gene symbol	Skull v Limb
Ankrd1	0.26
Emp1	2.15
Ephb2	3.49
Fkbp9	2.19
Fkbp11	2.12
Lepre1	2.55
Maged1	2.63
Mkrn1	0.33
Ogn	2.01
PCDH19	2.33
Ptn	2.13
Rcn	2.74
Rhced	0.32
Serpinh1	2.09
Timp1	3.04

### mRNA expression in primary adult bone-derived cells

Even in attempts to generate monocultures, cultures of isolated osteoblasts from bone tissues will contain other cell types. The cells used in these experiments are from third collagenase digests of adult bone and may thus contain osteoblasts along with osteocyte, osteoclast, endothelial, neuronal and adipocyte cells. As a precaution, genetic markers indicative of contamination with another tissue type were assessed. Markers selective for osteoclast, adipocyte, neurofilament, monocyte, muscle and endothelial cell were expressed, if at all, at low levels in the cultures. There was no significant difference in the expression of these markers between the S-Bdc and L-Bdc cultures (data not shown). The level of gene expression of osteocalcin was low.

**Figure 2:**
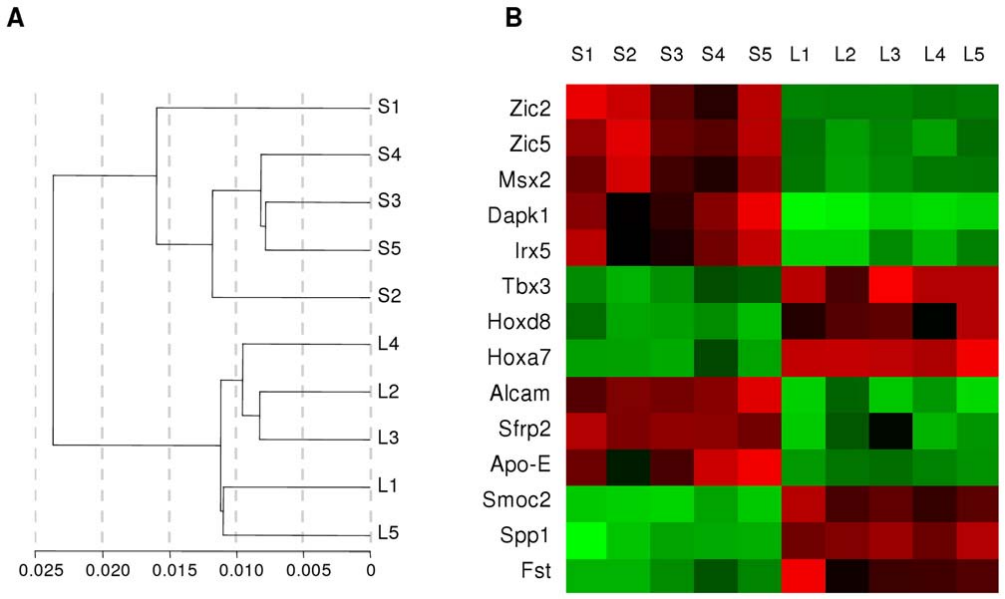
Genotypic differences between 5 matched pairs of skull and limb adult bone-derived cells. (*A*) Dendrogram signifying populations are distinct in isolated S-Bdc and L-Bdc. (B) Heat map for markers for regional development: cultured S-Bdc – (S1–S5) preferentially express genes associated with the craniofacial development of neural crest derived cells (red), whilst genes for limb development and patterning are preferentially expressed by L-Bdc (L1–L5). Differential expression of bone-associated markers are also depicted; Alcam, activated leukocyte cell adhesion molecule; Sfrp2, secreted frizzled-related protein 2; Apo-E, apolipoprotein E; Smoc2, SPARC related modular calcium binding 2; Spp1, osteopontin; Fst, follistatin.

249 genes were significantly and differentially expressed in S-Bdc and L-Bdc. This represents approximately 1% of the genome, and cluster analysis segregates these samples by their site of origin (Figure 2A). Again, markers for regional development and bone metabolism are differentially expressed site-specifically (Figure 2A, B). The absolute level of expression of markers associated with regional identity is low compared with bone differentiation markers, but the differential expression between sites is relatively large (3x–260x). Conversely, expression levels of bone differentiation markers are high, but differential expression differences are relatively small (3x–6x).

**Figure 3:**
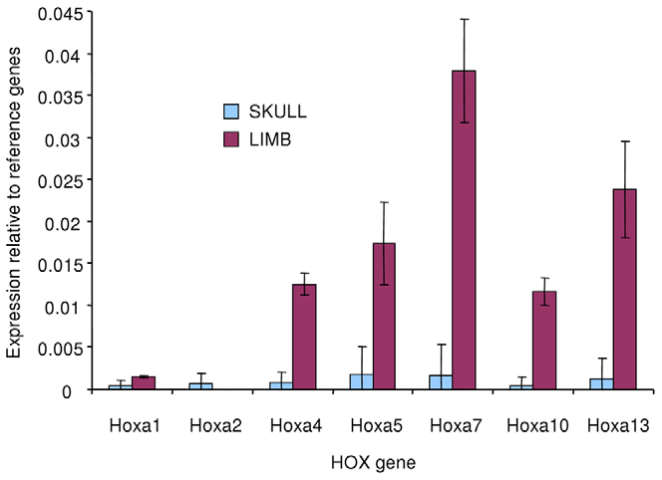
Site-specific *Hoxa* cluster gene expression of cultured adult bone-derived cells. Isolated osteoblasts derived from the skull and limb of adult rats maintain a specific patterning of *Hoxa* gene expression in culture.

Expression of *Hoxa* cluster genes in S-Bdc and L-Bdc were determined by qRT-PCR. Levels of all *Hoxa* genes are around detection threshold in S-Bdc compared with consistently higher levels of expression in L-Bdc. There was no significant difference between in the expression of *Hoxa2*, whereas significance levels were p<0.01 for all other comparisons (Figure 3).

**Figure 4:**
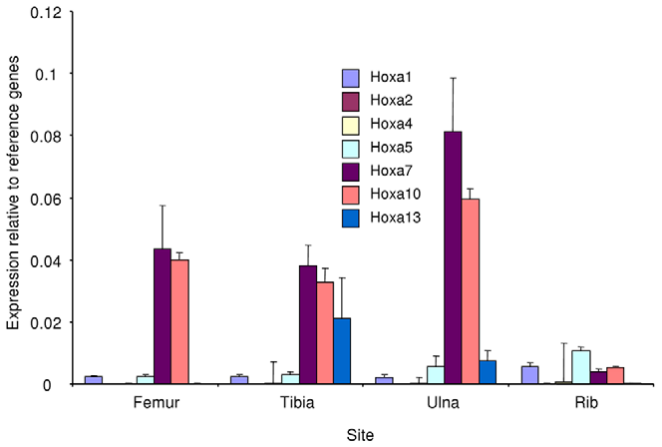
Site-specific *Hoxa* cluster gene expression profiles of cultured adult bone-derived cells. Continued expression of developmental patterning *Hoxa* genes demonstrate posterior prevalence in adult limbs; the more distal the skeletal element, the greater the *Hoxa* number expression. For instance, *Hoxa13* is expressed in the more distal ulnar and tibial bones, but not in the more proximal femur.

To distinguish whether this distinct pattern of *Hoxa* expression is seen in other endochondral bones, we compared *Hoxa* expression in osteoblasts isolated from other limb and axial (rib) bones (Figure 4). Compared with the appendicular bones, the relative expression of genes in the *Hoxa* cluster were lower in the rib, except for Hoxa2 and Hoxa5. Evidence for posterior prevalence in the adult is apparent; *Hoxa13* is not expressed in the femur samples whilst *Hoxa13* is expressed in tibial and ulnar bones which are the more distal skeletal elements.

Table 5 presents validation of the gene array techniques with qRT-PCR, and illustrates the close comparison of fold differences between gene array and qRT-PCR in a range of significant and differentially expressed transcription factors, receptors and matrix proteins.

**Table 5:** Validation of gene array with qRT-PCR (Skull/Limb ratio).

Sample	Method	Gene	Array	qRT-PCR
Bone organ	Affymetrix	CyclinD	3.92	2.97
		Shox2	0.08	0.09
		Ibsp	2.03	4.61
		Cdh1	0.40	0.32
		Lepre1	2.53	2.29
		Cnr2	0.42	0.19
Cells	Illumina	Hoxa7	0.02	0.04
		Irx5	16.10	6.95
		Msx2	13.10	11.40
		Reln	0.01	0.12
		Spp1	0.30	0.08
		Alpl	1.28	3.18
		Expi	0.21	0.13
		Bglap2	1.16	1.05
		Tbx3	0.13	0.41
		Tgfbi	0.20	0.11

The difference in the number of differentially expressed genes in bone (1236) and isolated cell (249) cultures may reflect the populations that comprise the two models. Whilst we have provided culture conditions that favour osteoblasts over other cell types, we accept other cell types may be present. However, in the bone organ we know there are more cell populations present within the samples (for instance, osteocytes, adipocytes, endothelial, neuronal-like hematopoietic cells). Notwithstanding, in either case, cluster analysis segregates skull and limb bone and isolated osteoblasts into separate populations.

### Skull and limb bone matrix phenotype

**Figure 5:**
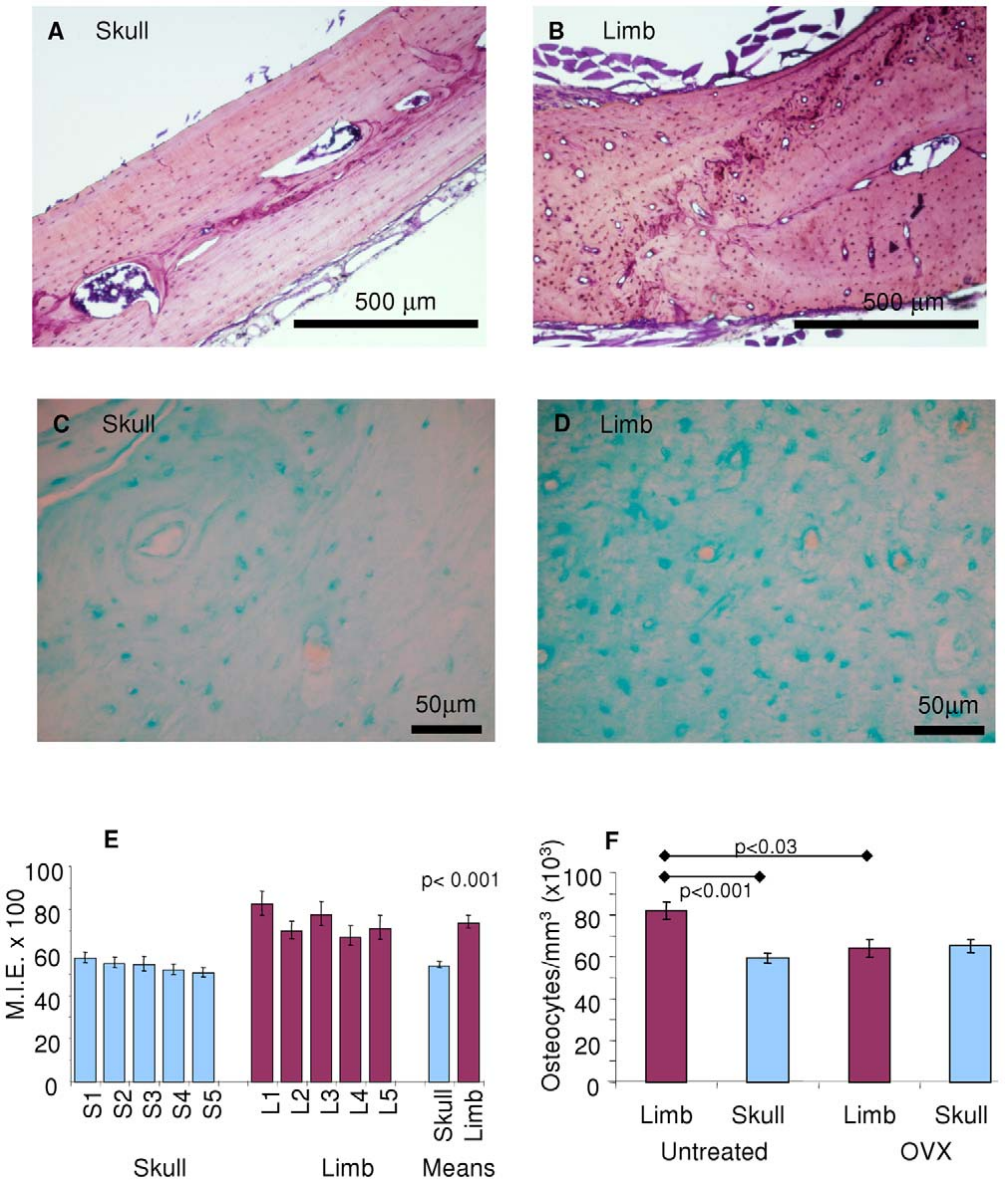
Phenotypic differences between skull and limb bone. Low power photomicrographs of (*A*) skull and (*B*) limb stained with Toluidine blue for histology and higher power (*C*) skull and (*D*) limb bone stained with Alcian Blue 8GX. Regions of acellular bone matrix were masked in the microdensitometer and measured. This ensured that the heavily stained osteocytes did not interfere with the readings. (*E*) Quantitative assessment by microdensitometry of polysulphated glycosaminoglycan levels in bone matrix stained with Alcian Blue 8GX. (*F*) Ovariectomy alters osteoblast incorporation into bone matrix as osteocytes in limb bone, but not skull bone, during growth.

Clearly the gross anatomy of skull and limb bones is distinct, but so too is the finer structure (Figure 5A and B). The concentration of polysulphated glycosaminoglycans in the osteoblast-derived bone matrix is significantly greater (36%) in limb bone compared with skull bone (Figure 5C, D and E). The number of osteocytes per unit volume of skull and limb bone differ significantly (40% greater in the limb, p<0.001, n = 5 matched pairs). A change in bone cellular composition was measured after 7 months of growth following ovariectomy. Osteoblast incorporation as osteocytes into bone matrix was reduced in limb bone, but remained unaltered in the skull vault (Figure 5F).

### S-Bdc and L-Bdc phenotype

**Figure 6:**
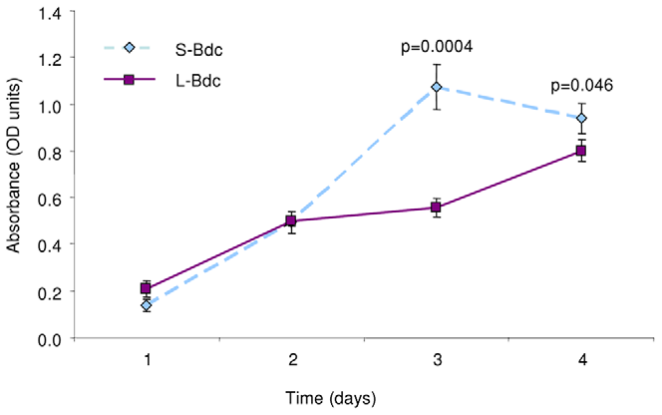
Adult bone-derived cell proliferation. Assessment of cell proliferation using MTS. Bone-derived cell populations from the skull proliferates more rapidly than limb bone derived cells.

**Figure 7:**
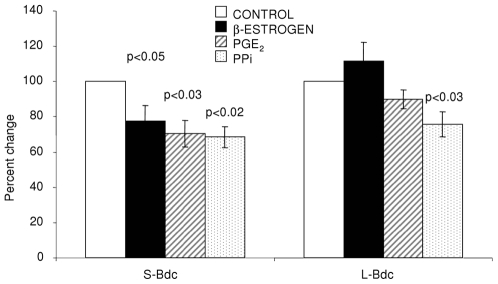
Effect of osteotrophic agents on ALP activity of adult bone-derived cells. Isolated adult bone-derived cells were seeded at equal densities and treated with either β-estrogen (10^−8^ M), Prostaglandin E_2_ (10^−6^ M) or inorganic pyrophosphate (0.5 mM) for four days. Data is presented as percent differences from control. Estrogen differentially regulates normalised ALP activity in S-Bdc and L-Bdc.

Adult bone-derived cells in culture were assessed for differences in proliferation rates and cell size. Direct cell measurements showed that mean cell volume and diameter measurements were greater (26%, p<0.02 and 8.5%, p<0.02 respectively, n = 5 matched pairs) for S-Bdc compared with L-Bdc (Table 6). Cells were plated at identical starting densities and showed significant differences in number (as judged by MTS assay) after 3 days in culture (S-Bdc > L-Bdc, p<0.004, n = 5 matched pairs), L-Bdc were still proliferating at day 4 (Figure 6). The effects of osteotrophic agents on alkaline phosphatase (ALP) activity were assessed in both S-Bdc and L-Bdc. Data was normalised to DNA content. Estrogen (10^−8^ M) decreased ALP activity in S-Bdc, but increased activity in L-Bdc (Figure 7). The number of mineralized nodule generation in culture was low (data not shown), and probably reflects the low levels of osteocalcin expression.

**Table 6:** Physical characteristics of S-Bdc and L-Bdc.

Parameter	*p*-value	Site	Mean	Std. Dev.
Diameter (mm)	<0.02	S-Bdc	19.49	0.55
		L-Bdc	17.96	0.77
Volume (fl)	<0.02	S-Bdc	4692	375
		L-Bdc	3719	473

## Discussion

Our analysis reveals that the matrices of these functionally distinct bones show measurable differences in composition. Glycosaminoglycan (Gag) concentrations are higher in limb bone. Osteocyte density in the limb bone was decreased in OVX animals, which is consistent with OVX-induced decreases in other non-cranial skeletal sites [33]. However, the osteocyte density in skull bone was unaffected during growth in an ovarian estrogen-depleted animal. This is consistent with differential responses of regional skull and limb osteoblasts and the pattern of bone loss induced by menopausal hormonal changes. However, we can not preclude that any decrease is partly in response to ageing [34].

Although we do not have direct evidence that the local patterns of gene expression in individual bones of the skeleton would determine site-specific characteristics [35] it is a distinct possibility. Differential expression of genes linked to osteoporosis in skull and limb bones is a powerful argument to mis-express these genes in limb bone in future studies. However, those genes differentially expressed in both bone organ and osteoblast cultures are more likely to establish these genes as potential therapeutic targets. Two such genes are detailed here, namely collagen triple helix repeat containing 1, (*cthrc1*) and reelin (*reln*).

cthrc1 has been implicated in increased osteoblast proliferation and higher bone mass; in isolated osteoblasts from *cthrc1*-null mice, alkaline phosphatase, collagen type 1α1 and osteocalcin expression levels were reduced [36]. Further, cthrc1 impacts on responsiveness to TGF-β and subsequently TGF-β target genes including collagens type I, α1 and α2 in PAC1 cells [37]. The canonical Wnt pathway is involved in the regulation of limb bone mass [38] but not development of the skull [39] and cthrc1 has been shown to suppress the canonical pathway and activate the planar cell polarity (PCP) pathway [40]. *cthrc1* expression was significantly greater in bone organ (2.8) and adult bone-derived cell (3.73) samples derived from the skull (2.83 and 3.73 times, respectively), suggesting Wnt/PCP-based signalling maybe more important in osteoblasts in the skull. We are unable, however, to detect any differential expression in the potential downstream osteotrophic targets in these adults.

Differential expression of several neurogenic markers was identified in bone samples. In particular, there was elevated expression of *reln* in limb bone (2–3 fold) and L-Bdc (7.7 fold, Table 5). *Reln* expression has recently been associated with abnormal bone remodelling of the otic capsule in the pathogenesis of otosclerosis [41]. The extracellular protein reelin is associated with axonal guidance and the basis of learning and memory via synaptic plasticity [42]. The significance of the neuronal-like appearance of the osteocyte network has been previously noted [43] and osteocytes have been proposed as mechano-sensors [43], [44] that communicate to maintain target strain with brain-like glutamate-based mechanisms [45]–[47]. Hypermethylation of the *reln* promoter is often seen during ageing and is associated with its reduced expression [48]. In the brain, reduced *reln* expression or reelin signalling activity is implicated in the generation of Alzheimer's disease [42]. It is tempting to speculate a role for osteocytic reelin [49] in the loss of mechanically-regulated limb bone remodelling and target strain maintenance in the aged. Measurement of the osteogenic response to applied mechanical loading of the *reln* knockout, reeler, mouse [50] would test the importance of reelin in limb bone biology. Interestingly, *reln* expression in skull bone and S-Bdc is significantly lower compared with limb bone and L-Bdc. This is consistent with skull bones possessing a distinct pathway independent of the mechano-adaptive system of the limb.

A third differentially expressed gene, with increased expression in skull bone (skull∶limb ratio 2.9) and S-Bdc (8.3), is *rerg* - a ras-like and estrogen-regulated growth inhibitor [51]. In manipulated HEK293 cells, *rerg* is a target gene of the estrogen receptor-β [52], there are as yet, however, no defined roles for *rerg* in bone cell biology. Whether the differential effects of estrogen on ALP activity (Figure 7) are modified by *rerg* has not been assessed.

Positional identity along the anterior-posterior body and proximal-distal axis of the limbs relies on the pattern of homeobox gene expression. *Hox* genes act in a hierarchical manner based on ‘posterior prevalence’ whereby the most posterior expressed *Hox* gene determines positional identity [53], [54]. This mode of patterning is reinforced by *Hox* gene/microRNA clusters that promote suppression of the anterior characteristics [54]. Expression of Hox genes could therefore be part of the mechanism that directs osteoblasts to function appropriately for their skeletal location [55].

To confirm that regional gene expression profiles are an inherent property, and not dependent upon the local mechanical environments, we investigated *Hoxa* gene expression profiles in adult bone-derived cells in a range of long bones and from the rib. Differential *Hoxa* expression profiles (*Hoxa2*, *Hoxa5*, *Hoxa7* and *Hoxa10*) are clearly evident in these endochondral/mesoderm bones. Investigations have not yet disclosed whether such regional gene expression differences underscore the differential responses to systemic anti-osteoporotic bisphosphonate treatment [56]. Concordant with posterior prevalence, *Hoxa13* is present only in the ulna and femur samples. Our observations agree with studies showing that cultured human skin fibroblasts express a specific HOX gene profile depending on the region of the body from where they are derived [57], [58].

Although *HOXA10* has been shown to regulate the expression of *RUNX2*, ALP and osteocalcin [59], attributing a direct function to the differential expression of *Hoxa* genes is difficult; site-specific cell behaviour and local *Hox* gene expression profiles may simply be correlated. However, in the adult, intramembranous-derived mandibular periosteal cells (*Hoxa11 negative*) can fill wound defects drilled into jaw and tibial limb bones. In contrast, periosteal cells from the endochondral-derived tibia (*Hoxa11 positive*) fail to repair the defect in the jaw; the transplanted cells differentiate into chondroblasts rather than osteoblasts [60]. These experiments suggest appropriate (source-dependent) function is correlated with *Hoxa11* expression. Phenotypic stability of osteoblasts is required for the replacement of the appropriate bone tissue following trauma and the remodelling process and increased expression of *Hox* genes have been described at fracture sites [61], [62]. Our data suggests that phenotypic stability is maintained in osteoblasts but that the appropriate site-specific behaviour may be more complex than being simply positive or negative for expression of *Hoxa11*.

The persistence of homeobox gene expression profiles in the adult mirror positional specification during embryogenesis and suggests that cells maintain a ‘memory’ of their origin and/or body positioning – an ‘epigenetic postcode’. These observations suggest a basis by which autonomous regulation of osteoblast behaviour at different sites and could explain in part how systemic hormonal changes differentially affect the skeleton. These findings support our contention that skull and limb bones form and are maintained by distinct cell (osteoblast) populations. Whether there is any correlation between the site-specific differences in osteoclasts [21], [22] and their homeobox expression profiles has yet to be investigated.

There is still controversy regarding the embryonic lineage of the parietal bone – whether it is mesoderm or cranial neural crest. Grafting and fate mapping experiments show distinct lineages compared with the use of Wnt-cre lineage tracing experiments. A report by Yoshida, clearly shows the lack of neural crest cell marker, X-gal staining in the parietal bones of Wnt1-cre/R26R mice [63]. This may reflect the discontinuity of Wnt1 expression along the neural tube, which shows a distinct gap in expression around the hindbrain/midbrain junction [64]. This gap in expression is approximately where neural crest that could contribute to the parietal bone would be expected to arise. Therefore it seems possible that at least some of the cranial neural crest may remain unlabelled which and could account for the lack of X-gal staining between the interparietal and frontal bones.

*Cart1* expression in skull bone is surprising since *cart1* is predominately expressed in the chondrocyte lineage (Table 2). However, *cart1* is known to be essential for normal skull bone development, null mice develop with normal limbs and trunk, but the interpatietal and majority of the parietal and frontal bones are absent [65]. It appears that during skull bone development a unique cell type, the ‘chondrocyte-like osteoblast’ is present [66]. Whether *cart1* is expressed by chondrocyte-like osteoblasts, and whether this determines the nature and characteristics of skull bone matrix has yet to be determined.

It has not been established whether the neural crest contribution to bone tissues has any clinical relevance – however, the clavicle may provide some useful insights to the development of skeletal disorders. Unusually, this bone develops by both intramembranous ossification (laterally) and endochondral ossification (medially) [67], and studies have demonstrated that only the medial aspect contains cells derived from the cranial neural crest [68]. The lateral aspect of the clavicle is prone to osteoporosis and is consistent with other bones containing a neural crest component (skull vault) being resistant to osteoporosis.

In conclusion, this study shows that genes associated with bone mass and mineral density are differentially expressed in functionally distinct skeletal sites. Our observations also demonstrate developmental gene expression-based “positional identity” in the adult skeleton. If positional identity is significant for bone then more favourable clinical outcomes would be expected with site matching of bone source to recipient site in grafts and tissue engineering/regeneration protocols. It may also be possible to exploit differential positional identity markers to develop site-directed pharmacological treatments. We propose that osteoblasts, and the matrix they produce, differ according to their location and that these differences are established, at least in part, by the developmental origin of the cells that contribute to the site-specific osteoblast lineage.

## Materials and Methods

### Ethics statement

All animal procedures were carried out in accordance with the UK Home Office Scientific Procedures Act (1986). All animals were purchased from Charles Rivers, housed, and fed *ad libitum* in accordance with local Queen Mary University of London, School of Medicine and Dentistry rules.

### Animals

Male rats were housed until they reached weights that matched those used in previous mechanical loading experiments [3]. Females were ovariectomised pre-pubertal (by the supplier) and maintained until 7 months of age prior to assessment of osteocyte number. The pilot studies used ten male skeletally mature Black CD57 (24–26 grams) mice divided into two groups to test for the feasibility of gene array in bone organs.

### Adult bone-derived cell isolation and culture

Five male CD rats (100–110 grams) were rendered unconscious with CO_2_ and killed by cervical dislocation. Skull and the cortex of ulnar bones were dissected and cleared of attendant soft tissues, epiphyses and marrow. Individual samples were minced and bone cells isolated by enzymatic digestion. Third digest cell populations were used in the experiments. 5 matched pairs of adult bone-derived cultures were thus isolated. Cells were cultured in αMEM (Gibco), 10% newborn calf serum (First Link), 1x penicillin/streptomycin and 1x fungizone) at 37°C, in a 5% CO_2_ atmosphere. Cells were fed every 2 days. Following passage, osteoblast numbers, cell volumes and size were determined using a CASY® (Model TTC) Cell counter and analyzer system. For proliferation assessment, matched pairs were grown in a series of 6-well plates. Plates were removed from the experiment on a daily basis and proliferation assessed using the Promega® CellTiter 96 Aqueous Non-radioactive Cell proliferation assay system. The MTS reaction product, formazan, was measured using a BMG Labtech FLUOstar OPTIMA plate reader at 490 nm. To assess ALP activity cells were washed in PBS and incubated with 12.5 mg *p*-nitro-phenylphosphate per ml of Sigma alkaline buffer No. 221 at 37°C for 20 minutes. The reaction was stopped using 0.5 M NaOH solution and the yellow reaction product read on a plate reader at 405 nm. DNA content was assessed according to methods determined by Rago [69]. Water-soluble 17β-estadiol (E4389, estrogen), Prostaglandin E_2_ (P5640) and sodium pyrophosphate (P8010) were obtained from Sigma.

### RNA extraction

Total RNA from skull and limb bones cleared of attendant soft tissues and epiphyses or sutures was extracted from five groups of four male CD rats (100–110 grams) using an RNeasy Fibrous Tissue Mini Kit (Qiagen) as per protocol. For isolated S-Bdc and L-Bdc, RNA was extracted from near confluent third passage cells collected from the ten populations using the RNeasy Mini Kit (Qiagen) as per protocol. RNA was extracted from osteoblasts isolated from other long bones using the RNeasy Mini Kit from first passage near confluent cultures.

### Microarray analysis

The Affymetrix Mouse Genome 430 2.0 GeneChip and Rat Genome 230 2.0 GeneChip were used to detail basal gene expression profiles in the bone organ samples. Biotinylated targets suitable for hybridization to the GeneChip probe arrays were prepared from the RNA samples as per ‘One-Cycle Target Labeling' protocol (Affymetrix). Briefly, double-stranded cDNA was synthesised from total RNA followed by *in vitro* transcription reaction to produce biotin-labelled cRNA from the cDNA. The cRNA was then fragmented prior to hybridization to the GeneChip arrays. Analysis of data was performed using GeneSpring 6.1 (Silicon Genetics, Redwood City, USA) software. The ‘per chip’ and ‘per gene’ normalization procedures, as recommended, were used. Statistically significant (ANOVA, p<0.05, Benjamini and Hochberg false discovery rate) gene lists representing differentially expressed genes, and expression tree were generated. The Illumina RatRef-12 Expression BeadChip was used to detail basal gene expression profiles in the primary cultured skull vault and ulnar osteoblasts. BeadStudio software was used to analysis the data. A ‘Diff score’ of <65 and >65 for gene expression for 5 matched pairs are considered as significantly different (p<0.05).

Gene array data is available on the NCBI Gene expression omnibus, accession number: GSE12966.

### qRT-PCR analysis

Gene array data was validated by qRT-PCR using Taqman Assay-On-Demand oligonucleotides for the following genes (Table 6). cDNA was prepared from 1 µg total RNA isolated from each the 10 bones or primary cell lines using oligo dT primers (Promega) and Promega reagents. The reaction conditions were 70°C for 5 mins (denaturation), 60 mins 40°C for 60 mins (extension), 70°C for 15 mins (inactivation), and then stored at −20°C. Each TaqMan assay was run in four replicates for each RNA sample. 50 ng total cDNA (as total input RNA) in a 10 µl final volume was used for each replicate assay. Assays were run with 2xAbsolute qPCR ROX Master Mix (Abgene) on Applied Biosystems 7900 Fast Real-Time PCR System using universal cycling conditions (10 min at 95°C; 15 s at 95°C, 1 min 60°C, 60 cycles). The assays and samples were analyzed on 384 well plates. Data normalization: in qRT-PCR an endogenous control gene is used to normalize data and control for variability between samples as well as plate, instrument and pipetting differences. Eif4a2 and ATP5b were chosen as the reference genes because their C_T_ values showed the least variation across the samples (data not shown). Each replicate C_T_ was normalized to the average C_T_ of Eif4a2 and ATP5b on a per plate basis by subtracting the average C_T_ of Eif4a2 and ATP5b from each replicate to give the ΔC_T_ which is equivalent to the log_2_ difference between endogenous control and target gene. When TaqMan gene expression assays are run on a 7900HT system in a 10 µl reaction volume, a raw C_T_ value of 34 represents approximately ten transcript molecules (assuming 100% amplification efficiency). At a copy number less than five, stochastic effects dominate and data generated are less reliable. Thus, a raw C_T_ of 35 was set as the limit of detection in this study: individual replicates which gave C_T_ values >35 were considered not detected. A C_T_ >32 and <35 (∼5–40 transcript molecules) was considered a low expressing gene. Differential expression of selected genes in both gene arrays and qRT-PCR are presented in Table 6.

### Histological preparations

Decalcified bone sections (10 µm) were cut on a microtome housed in a cryostat at −25°C (Bright's) and flash dried onto glass slides. Sections were stained overnight in 0.05% alcian blue 8GX solution in 0.025 M acetate buffer (Sigma), containing 0.025 M MgCl_2_ (Sigma) at a final pH of 5.8. A Vickers M85A scanning and integrating microdensitometer was used to quantify the intensity of Alcian Blue staining [70]. Osteocyte density was determined in similarly prepared sections. 10 images of equal and known area of bone per section were captured (Olympus BHS microscope linked to a Kontron image analysis system). Osteocyte number was counted in two sections per bone and presented as number per area.

### Statistics

Data is presented as means and significance tested using paired *t*-test, unless otherwise stated and p<0.05 was considered significant.
